# Immune Responses to *Pseudomonas aeruginosa* Biofilm Infections

**DOI:** 10.3389/fimmu.2021.625597

**Published:** 2021-02-22

**Authors:** Claus Moser, Peter Østrup Jensen, Kim Thomsen, Mette Kolpen, Morten Rybtke, Anne Sofie Lauland, Hannah Trøstrup, Tim Tolker-Nielsen

**Affiliations:** ^1^ Department of Clinical Microbiology, Rigshospitalet, Copenhagen University Hospital, Copenhagen, Denmark; ^2^ Costerton Biofilm Center, Department of Immunology and Microbiology, Faculty of Health and Medical Sciences, University of Copenhagen, Copenhagen, Denmark; ^3^ Department of Plastic Surgery and Breast Surgery, Zealand University Hospital, Roskilde, Denmark

**Keywords:** biofilm infections, *Pseudomonas aeruginosa*, innate immune response, adaptive immune response, novel treatment possibilities

## Abstract

*Pseudomonas aeruginosa* is a key pathogen of chronic infections in the lungs of cystic fibrosis patients and in patients suffering from chronic wounds of diverse etiology. In these infections the bacteria congregate in biofilms and cannot be eradicated by standard antibiotic treatment or host immune responses. The persistent biofilms induce a hyper inflammatory state that results in collateral damage of the adjacent host tissue. The host fails to eradicate the biofilm infection, resulting in hindered remodeling and healing. In the present review we describe our current understanding of innate and adaptive immune responses elicited by *P. aeruginosa* biofilms in cystic fibrosis lung infections and chronic wounds. This includes the mechanisms that are involved in the activation of the immune responses, as well as the effector functions, the antimicrobial components and the associated tissue destruction. The mechanisms by which the biofilms evade immune responses, and potential treatment targets of the immune response are also discussed.

## Introduction

Biofilms consist of microbes located in densely packed slow growing microcolonies embedded in a self-produced protective biopolymer matrix. In this life-mode, the microbes attain the highest levels of resistance to our present assortment of antibiotics and the immune system (1, 2). Accordingly, biofilms are a common cause of persistent infections (3), and biofilm-based infections are a major socio-economic burden implicating hospitalization, patient suffering, reduced life quality, increased mortality risk and lost employment. It is estimated that about 60%–70% of hospital acquired infections are caused by microbial biofilms (4). The immune response to biofilms results in collateral damage of adjacent tissues and therefore is an important aspect of biofilm infection pathology (5).

The vast majority of studies of the immune response against bacteria have focused on infections caused by bacteria in the planktonic state. Accordingly, considerably less is known about the immune response to bacteria growing in biofilm-based infections. However, recent *in vivo* and *in vitro* studies have begun to reveal the nature of both the innate and adaptive immune responses to biofilms (5, 6).

Planktonic bacteria are recognized by the innate immune systems pathogen recognition receptors (PRRs) through interaction with pathogen-associated molecular patterns (PAMPs), such as the flagellum and lipopolysaccharide (LPS) recognized *via* Toll-like receptor 5 and 4, respectively (7). Basically, biofilm growing bacteria activate the immune system through the same pathways as planktonic growing bacteria (5, 6). However, when residing in a biofilm the bacteria are embedded in extracellular polymeric substances and the classical PAMPs are less exposed to the immune system. In addition, PAMPs can be down-regulated in biofilm growing bacteria, as has been shown for flagella in *P. aeruginosa* (8, 9). Thus, in the case of biofilm infections the extracellular matrix components of the biofilms play an important role for the immune response (5, 6, 10).

The inflammatory state induced by biofilm unusually involves activation of both the innate and the adaptative immune response due to the chronic nature of biofilm-associated infections. Neither immune response is capable of eradicating biofilm, but they instead lead to extensive secondary damage.

**Figure 1:**
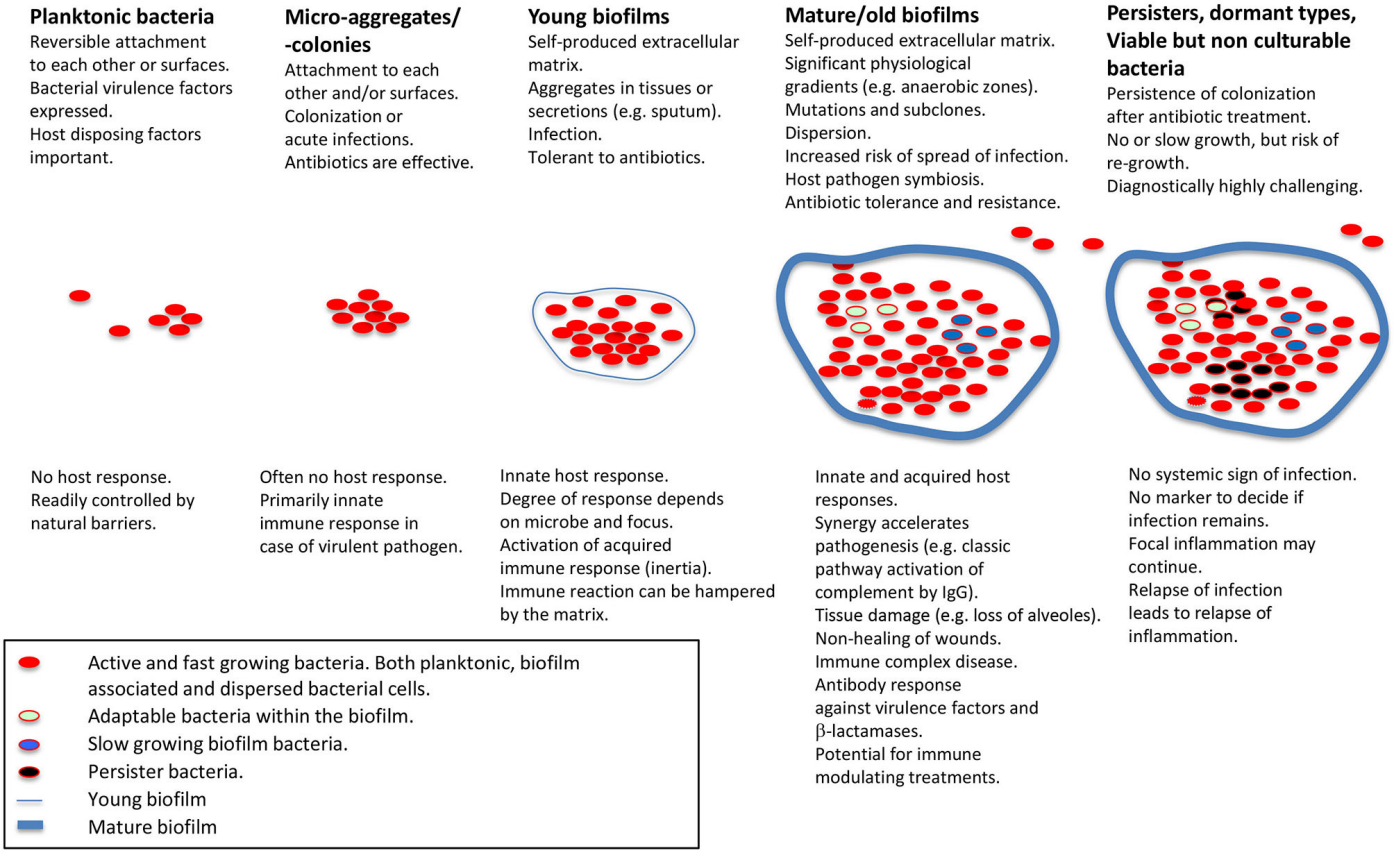
Schematic presentation of biofilm stages and host response. Applies for non-foreign body-related biofilm infections, which is the main focus of the present review. Modified from Moser et al. (5) with permission from John Wiley & Sons, Inc.

The present review is focused on interactions between *P. aeruginosa* biofilms and the immune system (**Figure 1**). *P. aeruginosa* is involved in several persistent biofilm infections, including cystic fibrosis (CF) lung infections, chronic wound infections, urinary tract infections with or without catheters, and tracheal tube related ventilator-associated pneumonia (11–13). These infections are difficult or impossible to eradicate with antibiotics alone due to the special physiological state of bacteria in biofilms (2). The immune response has detrimental effects, as it causes destruction of the lungs of CF patients and maintains the inflammatory state of chronic wounds (11, 14). Knowledge about the mechanisms involved in activation, regulation, and evasion of the immune responses, as well as the nature of the antimicrobial components produced by the immune cells, and the associated tissue destruction has increased in recent years and will be discussed in the present review. Organ-system specific immune responses can differ substantially due to significant differences in tissue anatomy and physiology and is discussed when appropriate. Measurement of adaptive immune response during chronic persistent infections has proven an important clinical tool and will be described. Even though the role of the adaptive immune response has long been well recognized as being crucial during healing of wounds and in particular in inflammatory skin disease, the study of the role of the adaptive immune response in chronic wounds with *P. aeruginosa* biofilm infection has only just recently taken off (15, 16). Therefore, we have not included a detailed description of *P. aeruginosa* biofilm in chronic wound infections in the section of adaptive immune response. The understanding of all these components of host responses during biofilm infections may eventually form a basis for development of new and effective treatments against biofilm-based infections.

## Biofilm Formation of *P. aeruginosa* During Chronic Infection

Biofilm formation by *P. aeruginosa* occur along with the production of several extracellular matrix components such as type IV pili (17–19), Cup fimbria (20), exopolysaccharides (21–23), CdrA adhesin (24), extracellular DNA (25), LecA/LecB lectins (26, 27) and Fap amyloids (28). The selection during chronic infection of *P. aeruginosa* variants that over-produce some of these biofilm matrix components is strong evidence for the involvement of biofilms in chronic infections (9, 29–32). Moreover, the presence of biofilms in CF lungs and chronic wounds has been demonstrated by microscopy (33, 34). *P. aeruginosa* can synthesize three different exopolysaccharides designated Pel, Psl, and alginate, although some strains only produce a subset of these exopolymers (21–23, 35). Overproduction of alginate enables mucoid *P. aeruginosa* strains to form persistent infections in the lungs of cystic fibrosis (CF) patients (29). Moreover, *P. aeruginosa* rugose small colony variants that overproduce Psl and Pel exopolysaccharide show enhanced persistence in CF lungs (9, 30, 31), and in chronic wounds (32). Evidence has been presented that Psl protects *P. aeruginosa* from host defenses in the initial phase of infection of the CF lung (36). Thus, it is possible that an extracellular biofilm matrix dominated by Psl is important in the initial stage of chronic lung infection before the bacteria mutate to produce a biofilm matrix dominated by alginate.

The host immune response plays an important role in the course of biofilm infections, and substantially affects the environment faced by the bacteria. The initial response to the presence of pathogens is an accumulation of activated neutrophils that may reduce the local O_2_ concentration due to O_2_ consumption accelerated by the respiratory burst and the production of reactive O_2_ species (ROS) and nitric oxide (NO) (37–39). Thus, O_2_ consumption by the neutrophils may result in O_2_ depletion in infected parts of the body (40). The restricted O_2_ availability accelerates stratified growth in *P. aeruginosa* biofilms, resulting in low metabolic activity in the center of biofilm as a consequence of nutrient depletion. However, micro-oxic conditions are sufficient to support growth of *P. aeruginosa* due to a highly flexible respiratory apparatus (41, 42) Moreover, bacteria may obtain energy under the anaerobic conditions prevalent in biofilm infections *via* anaerobic respiration or fermentation (43). Anaerobic respiration can occur by denitrification, where nitrogen oxides are utilized as alternative terminal electron acceptors (44, 45). The source of these N-oxides is suggested to originate from the rapid reaction of NO and O_2_ produced by activated neutrophils (44) resulting in the formation of peroxynitrite (ONOO^-^) (46), which may dismutate to nitrate $\left(NO_{3^{-}}\right)$ and nitrite $\left(NO_{2^{-}}\right)$ (47). The concentration of $NO_{3^{-}}$ and $NO_{2^{-}}$ in CF sputum (43, 48–50) may support *P. aeruginosa* growth at rates similar to those found in CF pulmonary biofilm (45). These findings suggest that the growth rate of *P. aeruginosa* during chronic CF lung infection is determined primarily by the number of surrounding neutrophils (51) which deplete O_2_ and produce $NO_{3^{-}}$ and $NO_{2^{-}}$ which can be used by the bacteria for anaerobic respiration. As biofilm formation, neutrophil accumulation and O_2_ depletion are common factors in multiple chronic infections, this interaction between host cells and pathogen is likely to occur also in other infections (44).

## Innate Immune Responses During *P. aeruginosa* Biofilm Infections

Innate immunity fights infections from the moment of first contact and is composed of germline-encoded, non-clonal cellular and humoral mechanisms. These mechanisms enable nonspecific defense against pathogens without former interactions with infectious microbial invaders (52). The main components of the innate immune response engaged in response to *P. aeruginosa* biofilm include neutrophils, macrophages, dendritic cells, NK cells, and the complement system.

The most solid demonstration of a role of innate immune responses to bacterial biofilm has been obtained by introducing human neutrophils and macrophages to *P. aeruginosa* biofilms devoid of planktonic bacteria (53–56). The observed response comprises neutrophil accumulation, respiratory burst, penetration, phagocytosis, production of cytokines and eradication of the biofilm bacteria. In addition, *P. aeruginosa* cultures with increased bacterial aggregation induced stronger respiratory burst by neutrophils and cytokine release by macrophages (57).

Likewise, early sampling of mouse lungs challenged with *P. aeruginosa* biofilms has shown that the innate immune response involves intense accumulation of activated neutrophils in the airways (54, 56, 58–60). Early accumulation of neutrophils at the site of *P. aeruginosa* biofilm infection is also evident from experimentally infected chronic wounds in mice (14).

## Innate Immune Response in CF Patients With Chronic *P. aeruginosa* Lung Infection

The innate immune response has gained particular attention in patients with CF and chronic *P. aeruginosa* lung infection, due to the association between accumulation of neutrophils in endobronchial secretions and reduced functionality of the lungs (61). The recruited endobronchial neutrophils display inflammatory activity as indicated by continuing respiratory burst (37, 62) and generation of nitric oxide (44). Accordingly, destruction of the lung tissue has been correlated with oxidative and proteolytic lesions of endobronchial neutrophil activity (63, 64). Chronic lung infections in CF patients are associated with defective apical ion transport due to mutations in the gene encoding the cystic fibrosis transmembrane conductance regulator (CFTR) (65). Infected CF lungs are dominated by *P. aeruginosa* growing as endobronchial biofilms surrounded by numerous neutrophils (33) and scarce planktonic bacteria, which are subject to phagocytosis by neutrophils (33, 37). The neutrophil response in infected endobronchial secretions in CF resembles the response in experimental *in vitro* and *in vivo* biofilms, where high numbers of neutrophils accumulate close to the biofilm (33) and depletion of molecular oxygen (O_2_) is accelerated (37). This is caused by the reduction of O_2_ to superoxide $\left(O_{2^{-}}\right)$ during the neutrophils’ active respiratory burst (66). Thus, the response of neutrophils to biofilms during chronic lung infection in CF may contribute considerably to the O_2_-depletion in infected CF lungs (40). Furthermore, as active neutrophils primarily rely on ATP generated by anaerobic glycolysis (67), the high intake of glucose by neutrophils in CF lungs (68) as well as the enhanced level of L-lactate in sputum from CF patients with chronic *P. aeruginosa* lung infection (69), is in agreement with a high activity of neutrophils during biofilm infection in CF lungs. The neutrophil response to planktonic *P. aeruginosa* likewise includes stimulation of the respiratory burst (37), suggesting that neutrophil activation may also include a response to planktonic *P. aeruginosa* in infected CF lungs. Moreover, activation of neutrophils in infected CF airways may be triggered by alginate (70), LPS or immune complexes (71). The intensity of the neutrophil response may be enhanced by priming with LPS (72) and soluble factors of the innate immune response, such as platelet-activating factor, TNF-α, IL-8 and leukotriene B4 (73–77). Additionally, the migration through inflamed tissue may lead to stimulation of neutrophils due to multiple engagements of integrins and inflammatory cytokines (78). The presence of infectious agents is actually not needed to stimulate the respiratory burst, as seen in response to injury of the intestine in mice (79). The apparent lack of significantly disturbed capacity of neutrophils in CF patients (76) suggests that the reaction of neutrophils to *P. aeruginosa* biofilms seen in CF patients may also apply to infectious *P. aeruginosa* biofilms in non-CF patients. Accordingly, biopsies from chronic wounds have revealed biofilm surrounded by high numbers of neutrophils (60, 80–82). Similarly, neutrophils accumulate in high numbers at infectious biofilm in prosthetic knees (83, 84), and the accumulation of neutrophils was intensified and prolonged by *P. aeruginosa* biofilms in experimental chronic wounds and peritoneal infection (14). Thus, the induction of the biofilm life style observed during interaction between *P. aeruginosa* and neutrophils *in vitro* (85–87) may be highly relevant for the formation of biofilm *in vivo*.

The capability of the innate immune system to recognize invading microorganisms is aided by PRRs that recognize and bind to conserved microbial PAMPs leading to stimulation of the host response. Numerous varieties of PRRs, and their matching ligands are known, but PRRs reacting with PAMPs specifically expressed in microbial biofilm have not been described. PRRs may exist as intra- and extra-cellular membrane-bound receptors, cytoplasmic receptors, or soluble receptors. Since their discovery Toll-like receptors (TLRs) have advanced to become a very well-known family of PRRs. One group of TLRs is expressed on the surface of host cells where they mainly recognize microbial membrane components including lipoproteins, proteins and lipids, while other TLRs are intracellular and recognize microbial nucleic acids (88).

**Figure 2:**
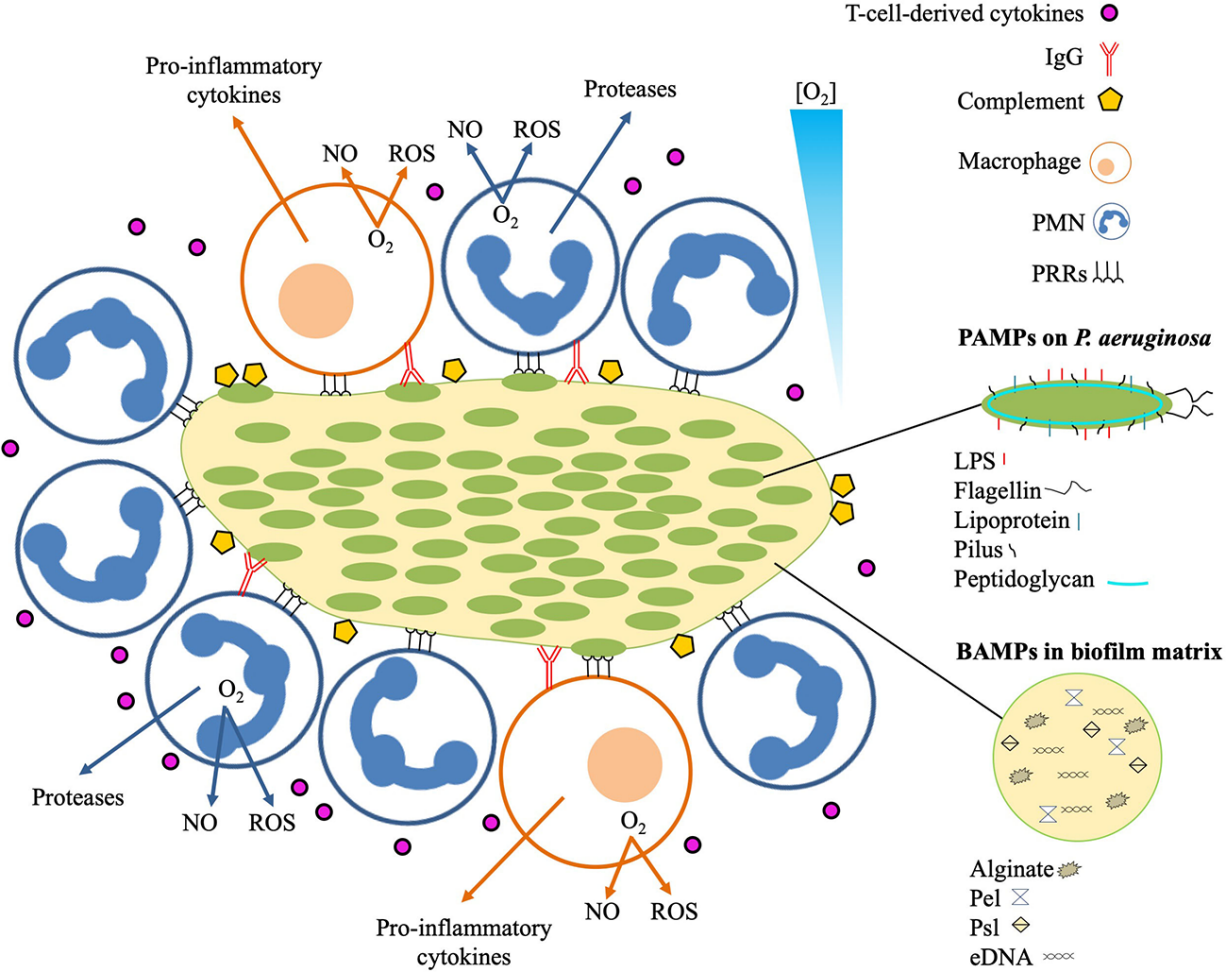
Local immune response to infectious *P. aeruginosa* biofilm. The innate immune response recognizes pathogen associated molecular patterns (PAMPs) expressed on *P. aeruginosa*, and biofilm-associated molecular patterns (BAMPs) present in the biofilm matrix. Detection of BAMPs and PAMPs by PMNs and macrophages is mediated by pattern recognition receptors (PRRs). Binding of BAMPs and PAMPs to PRRs stimulates the PMNs and macrophages resulting in consumption of O_2_ for liberation of tissue-toxic reactive oxygen species (ROS) and nitric oxide (NO). Additional responses by the PMNs include secretion of proteases that may cause proteolytic tissue lesions while the macrophage may further enhance the inflammation by emitting pro-inflammatory cytokines such as TNF-α, Il-1, IL-6, IL-8, and IL-12. The effector cells of the adaptive immune response mainly reside distantly such as the T-cells and the B-cells in the secondary lymphoid organs and the plasma cells in the bone marrow. Activated T-cells may release cytokines that further reinforces the inflammation by stimulating the accumulation and activation of PMNs and production of IgG. The contribution of the increased accumulation of activated PMNs to the local inflammation is further accelerated by binding of antigens to IgG, leading to immune complex mediated stimulation of the PMNs and activation of the classical complement pathway.

In the airways of chronically infected CF patients, TLR5 was the only MyD88-dependent TLR that was increased on neutrophils (89). This increased expression is possibly facilitated by G-CSF, IL-8 and TNF-α, and by the interaction of bacterial lipoprotein with TLR2 and TLR1 (88). TLR5 is a flagellin receptor (90) and its augmented expression on neutrophils in CF lungs is challenging to explain since flagella are lacking in mucoid biofilms *P. aeruginosa* isolated from CF lungs (91). The absence of flagella in nonmucoid biofilms, however, intensifies the bactericidal activity of neutrophils *in vitro* due to release of bactericidal amounts of lactoferrin (92), which may prevent biofilm formation (93, 94). Even though the significance of TLR5 expression for the outcome of biofilm infections is unclear, it may reinforce phagocytosis of planktonic, flagellin-intact *P. aeruginosa* subpopulations in the CF lungs (94). In support of this, neutrophils only ingested planktonic bacteria in infected airways of CF patients (33, 37), and *P. aeruginosa* with dysfunctional flagella survived for longer time during lung infection in mice (95). The capability of planktonic *P. aeruginosa* to provoke a stronger TLR-mediated response than biofilm *P. aeruginosa* has also been observed for the expression of IL-8 by epithelial cell lines (96). Bacterial eDNA, which is a matrix constituent of biofilms (25, 97), may stimulate neutrophils without involving TLR9 resulting in increased IL-8 production and intracellular signaling (98, 99). Alginate is an abundant component of the matrix in biofilm formed by mucoid *P. aeruginosa*, and is regarded as the strongest virulence factor in chronic lung infection in CF patients (100). Alginate may increase the respiratory burst of neutrophils (101), and monocytes may respond to alginate by initiating the production of cytokines (102). The activation of monocytes by alginate generated by *P. aeruginosa* may be mediated by TLR2 and TLR4 (103), while the PRRs involved in the activation of neutrophils remain elusive. The matrix of *P. aeruginosa* biofilms may contain other polysaccharide components, such as Psl and Pel, which may stimulate an innate response to biofilm (104). Recent evidence suggests that the specific exopolysaccharide composition of *P. aeruginosa* biofilms is a determinant of the neutrophil response (10). A biofilm with a matrix composed primarily of Psl and alginate polysaccharides was found to be particularly efficient in activating neutrophils (10). It remains, however, to be determined if the innate response against exopolysaccharide expression in biofilm is distinctly stronger than the innate response against exopolysaccharide expression in planktonic cells. In that case, we suggest considering exopolysaccharide as a subgroup of PAMPs termed “biofilm associated molecular patterns” (BAMPs) (**Figure 2**).

Although the soluble and the membrane-bound receptors of the complement system are among the most studied PRRs, a pivotal role of the complement system for the outcome of biofilm infections remains to be firmly established. Infectious biofilm may establish in spite of complement activation even in patients with intact complement systems. In this respect, *P. aeruginosa* may secrete elastase and alkaline protease that inactivate the complement system (105). Further protection may be provided by alginate with O acetylation which prevents complement opsonization of mucoid *P. aeruginosa* biofilms (106). The involvement of the complement system in CF lung infections has been demonstrated by the frequent isolation of activated complement (C3c) in the sputum from chronically infected CF patients (107). Furthermore, the matrix polysaccharide, Psl, protects mucoid bacteria from opsonization and killing by complement components in human serum (108). However, whether complement activation requires biofilm formation is unlikely since planktonic bacteria induce stronger activation of the complement system (109). However, *P. aeruginosa* isolated from CF sputum may escape activated complement system (110).

The intense buildup of neutrophils associated to *P. aeruginosa* biofilm infections in CF, chronic wounds and implanted devices, would be anticipated to eliminate the biofilm. However, specific defects may weaken the immune defense. Thus, as a consequence of the basic defect in CFTR, both neutrophils and macrophages in the CF lungs exhibit blunted phagocytic capacity that could contribute to poor bacterial clearance and altered efferocytosis (111, 112). Moreover, the failing bactericidal activity of the summoned neutrophils may rely on rhamnolipids produced by *P. aeruginosa* (56). Synthesis of rhamnolipid depends on quorum sensing (QS) (60) indicating the ability of *P. aeruginosa* biofilm to contain bacterial densities necessary to achieve the quora required to activate QS-dependent rhamnolipid production (56, 59, 60) in chronic wounds (81) and lungs of infected CF patients (33). Rhamnolipids protects the biofilm against approaching functional neutrophils by inducing cellular necrosis (60). Intriguingly, the molecule OdDHL may attract neutrophils (113) and may thus attract and lure the neutrophils to the site of infection where they are killed by rhamnolipids. The QS-regulated attenuation of the host response may facilitate the initial establishment of biofilm infection (6). However, succeeding lung infection in CF patients involves extensive genetic adaptions with frequent mutations, e.g. in the QS regulator gene *lasR* (114). Dysfunctional QS of the *lasR* mutants may result in defective proteolytic neutralization of chemotactic cytokines allowing the pro-inflammatory cytokines to attract increased numbers of neutrophils to the lungs leading to intensified pulmonary inflammation (115). The size of bacterial aggregates may also contribute to the protection of bacteria against the immune response offered from biofilm formation. In fact, when the size of aggregated *P. aeruginosa* with deficient QS exceeded diameters of 5 µm, phagocytosis by human neutrophils was inhibited (116).

*P. aeruginosa* in biofilms can produce additional virulence factors, such as pyocyanin, that may cause cellular damage and immune modulations in cystic fibrosis lungs (117). Pyocyanin has been associated to broader functions, such as impairment of ciliary beat frequency and mucin hypersecretion, which in turn create a positive loop for biofilm formation and dysregulated immune responses in the CF lung (118).

It may be expected that the infectious biofilm in CF lungs would succumb due to the potent antibiofilm activity of antimicrobial peptides produced by neutrophils and lung epithelial cells (119). However, the low pH in CF lungs may impair the antimicrobial activity of antimicrobial peptides (120, 121). In addition, the defective distribution of salts in CF lung may have crucial effect on the optimal functionality of some antimicrobial peptides (122). Other environmental conditions in CF lungs may contribute significantly to the reduced activity of antimicrobial peptides. These conditions include proteolytic degradation of antimicrobial peptides by bacterial proteases (123) and by host proteases (124) and inhibition of antimicrobial peptides by binding to complexes of LPS, F-actin, mucins, and host derived DNA (125).

## Innate Immune Response to *P. aeruginosa* Infection in Chronic Wounds

Whereas the majority of our knowledge on immune responses to *P. aeruginosa* biofilms comes from studies of CF lung infections, studies of chronic wound infections has recently shed additional light on the topic. The prevalence of recalcitrant wounds is expanding epidemically alongside with obesity and lifestyle diseases. The host response to bacterial intruders in chronic wounds is hallmarked by a persistent inflammatory phase. This phase comprises continuous oxidative damage, senescence of fibroblasts and skewing of constructive growth factors required for tissue resolution. The pathoetiology also includes low mitogenic-activity, high protease combined with low inhibitor-activity, microbiota changes, the etiology behind the original insult and the specific invading pathogen. Accumulating evidence emphasizes the paramount impact of infectious bacterial biofilm on the host response in the wound and the implication for recovery.

Unfortunately, it is challenging to achieve appropriate numbers of participants for conducting randomized studies on intervention in patients with recalcitrant wounds due to different chronicity definitions and patient heterogeneity. In addition, it is not feasible to extrapolate the results from chronic wounds of one etiology to another since many patients may suffer from several diseases (126).

The impact of infection with *P. aeruginosa* on wound chronicity is well described in clinical settings and experimental models (80, 81, 127). The presence of biofilm is now commonly recognized as a leading cause of chronic infections with persisting pathology despite antibacterial therapy and continuous induction of the host response (128). Certain components of *P. aeruginosa* biofilms, such as rhamnolipids, are likely playing important roles for persistence of infection as it causes cellular necrosis and killing of neutrophils (56, 59, 60). Other studies support the capability of *P. aeruginosa* to attenuate bactericidal components of the host defense (53, 129)

The endogenous antimicrobial peptides (AMPs) are phylogenetically ancient and constitute a crucial part of the skin’s innate defense to infection (130). AMPs may be made by keratinocytes and infiltrating granulocytes and macrophages in response to infection, wound healing, trauma, or chronic inflammation. In addition, AMPs possess regenerative properties (131). AMPs are amphipathic molecules (132), which enables interaction with phospholipids of microbial membranes leading to pore formation and bactericidal cell lysis (133). The endogenous antimicrobial host defense protein S100A8/A9 belongs to the alarmin group and displays various activities. S100A8/A9 is expressed in actively healing wounds in human and murines (134, 135), but S100A8/A9 is absent in chronic, colonized venous leg ulcers in humans (136, 137) possibly resulting from the distorted local host response. This is suspected to cause deterioration of wound healing.

Relevant animal models are valuable tools for obtaining knowledge on the interplay between host and pathogen. Accordingly, animal models have enabled detailed descriptions of disposing factors, infectious agents and host response to infection. There are obvious limitations when comparing murine to humane wound healing and regeneration. Mice heal with predominantly contraction in a looser attached skin with higher hair density and thinner dermis versus the humane granulation healing. There are also significant differences in the immune response, with more neutrophils in the humane circulating blood versus a higher number of lymphocytes in mouse blood in addition to substantial differences with regards to the antimicrobial peptides. Despite this, mice represent a generally accepted experimental animal of choice.

To study the interaction between biofilm and the host response, we have established a chronic wound model which enables examinations of *P. aeruginosa* biofilm-infected wound closure in two mouse strains. One strain is relatively resistant to *P. aeruginosa* infection and consists of C3H/HeN mice. The other strain is made up of BALB/c mice which are susceptible to the infection (14, 138). The C3H/HeN mice have Th1-dominated response towards the infectious agents *Leishmania* major and *Candida* species. On the contrary, the response of the BALB/c mice against these agents is Th2-dominated. The direction of the Th response has essential effects on mortality rates and clearance of infection (138). A dichotomized early response in the mouse model of chronic wounds has been indicated by the attenuated local IL-1β inflammatory response to *P. aeruginosa* biofilm during the first 5 days of infection in C3H/HeN mice as compared to the BALB/c mice (14). Furthermore, our group recently demonstrated that *P. aeruginosa* biofilm may decrease the intensity of local neutrophil response in several murine wounds which may compromise the control of infection. The connection between the slow healing and the genotype in BALB/c mice has been confirmed by another group (198), which makes this strain of mice an excellent choice of animal model for wound healing. In this context, comparing the spontaneous healing of *P. aeruginosa* biofilm infected wounds in C3H/HeN and BALB/c mice with the S100A8/A9 expression, could be highly valuable for further evaluation of the significance of S100A8/A9.

## Adaptive Immune Responses During *P. aeruginosa* Biofilm Infections

The adaptive immune system discriminates the host proteins and other potential antigens from foreign molecules, to ensure that the lymphocytic and humoral antibody mediated effector functions do not result in excessive damage to the infected organism. However, the adaptive immune reaction is extensively superior in the specific response, as compared to the innate responses. Furthermore, recognition of the identical or similar pathogen upon reinfection by the adaptive immune system advances rapid clonal expansion of up to a 1000-fold antigen specific effector and central memory cells at subsequent exposures. The developed memory is the premise for immunity to subsequent infections. Compared to innate responses, which cannot discriminate between primary and secondary responses, the secondary responses of the adaptive immune system is substantially faster, more potent and with enhanced affinity as compared to primary exposure (139, 140). Activation of the adaptive immune system often results in clearance of the infection by planktonic bacteria, due to the combined activity of the innate and adaptive immune systems augmenting both the immune reactions. However, in the case of chronic biofilm infections the pathogens are not eliminated. Instead, the synergy of the innate and adaptive immune mechanisms, the latter with inertia at first encounter, is a central component of biofilm pathogenesis (5, 141–143).

Activation of the adaptive host responses is facilitated through dendritic cells (DC) required for sufficient activation at the first pathogen encounter and macrophages (Mφ) (144). Immature DCs in the peripheral tissue are effective in antigen uptake and are especially abundant at pathogen exposed regions, as the mucosal surfaces and in the secondary lymphoid tissue (145, 146). DCs mature following antigen uptake, and from inflammatory cytokine impact, into mature DCs dedicated in antigen processing and presentation (145, 146). Therefore, the DCs are essential in linking the innate and adaptive immune systems, and have the exclusive capacity to prime naïve T-cells into subsequent Th1, Th2, or Th17 cells and responses (145–147). Due to the limited presence of DCs in tissues, isolation is highly challenging, especially in human studies. Our own studies using a chronic *P. aeruginosa* lung infection model revealed commitment of pulmonary DCs during the infection (148). Pulmonary DCs was demonstrated as early as 2 days of initiation onset (148). Interestingly, an increased number of DCs in the regional lymph node was not detected until day 7 (148). The fraction of activated pulmonary DCs increased during the 10-day observation period, when demonstrated by CD80 and CD86 expression (148). In contrast, the percentage of activated DCs in the lymph node decreased at day 10 (148). The cytokine release of the DCs from the lung and lymph node were in general paralleled. Interestingly however, the initial release of the pro-inflammatory cytokines IL-6 and IL-12 reached a maximum at days 2–3, followed by an increased IL-10 production at day 7 (148). This observation, likely represents an essential controlling role of the DCs in induction of the adaptive immune system effector functions, impacted by the adjacent innate responses (148). This is supported by observations from another study, where *P. aeruginosa* QS signal molecules diminished the murine DC IL-12 production, while the IL-10 release remained. In addition, antigen specific T-cell proliferation was down regulated by QS exposed DCs. These results indicates that DCs are inhibited in T-cell stimulation by the *P. aeruginosa* QS signals, and by this mechanism contribute to the *P. aeruginosa* biofilm pathology (6, 149).

From previous observations of GM-CSF and G-CSF on DCs, we hypothesized that the increased G-CSF would impact the DC response in chronically pulmonary *P. aeruginosa* infected CF patients, besides recruiting PMNs from the bone marrow (150). Indeed, the GM-CSF/G-CSF ratio and the IFN-γ response correlated, and interestingly also correlated to a better lung function. In contrast, IL-3 and IFN-γ responses correlated inversely (150–156). DCs seem to impact host responses in biofilm infections and represent a potential therapeutic target.

As mentioned above, the innate and adaptive immune effector elements function in collaboration. As a consequence of the persistent biofilm infection, the adjacent tissue is impacted by the injurious oxidative radicals and enzymes originating from the inflammatory cells. Besides the pathogen related virulence factors, elastases, proteases, and other exoenzymes resulting from the inflammation expedites degradation of crucial surface molecules of the immune cell, further adding to impaired anti-biofilm mechanisms of the host responses (107, 157–160). The ineffective host response is considered the key basis of the biofilm related pathology, since antibodies against several bacterial virulence factors, such as elastase, lipopolysaccharide, and flagella have been reported, which presumable should improve biofilm outcome (161–163). However, these virulence factors are considered to be involved in pathogenesis, predominantly during the initial phases and to support development from microbial colonization to infection *per se*. Although, the bacterial virulence factors are less involved in the direct chronic biofilm pathology, the antibody mediated precipitation of virulence factors and other microbe antigens results in formation of immune complexes deposited in the tissues. Since, this leads to activation of the complement system and PMN opsonization, tissue damage is the consequence (100).

A special situation of the adaptive immune response and chronic *P. aeruginosa* infection of airways is the induction of a mucosal antibody response represented by specific secretory IgA (sIgA). The IgG responses can be regarded as an element of the systemic immune response, and primarily get access to mucosal surfaces through inflamed epithelium. In contrast, sIgA is the primary antibody of mucosal surfaces, and it is produced in double the amount of IgG, and is secreted to the mucosal surfaces as dimeric sIgA bound to the secretory component (164). At the surfaces, sIgA functions through immune exclusion by binding to the pathogen and its PAMPs without activation of complement and opsonization. In CF sIgA has been found in sinuses and correlating to chronic sinusitis, whereas IgG dominates in the lower airways, where it correlates to inflammation of the respiratory airways (165). sIgA was also found to correlate to an early detection of *P. aeruginosa* of the lower airways of CF patients (165).

## T-Cell Response and Clinical Outcome in CF Patients With Chronic *P. aeruginosa* Lung Infection

The biofilm infection and host response interplay has been best characterized for CF patients with pulmonary chronic *P. aeruginosa* biofilm infections (6). Early intensive antibiotic therapy, maintenance antibiotic treatment strategy between exacerbations, and planned elective intravenous antibiotic courses has become standard of care in CF (11). However, the natural course of the pulmonary chronic *P. aeruginosa* infection revealed a dichotomized outcome. A poor outcome, and a pronounced or rapid escalation in antibody response, was reported for most CF patients (166). However, for a small group of CF patients the humoral response was modest and these patients had a beneficial outcome (166). In addition, the intensified antibiotic treatment strategy in CF, resulting in significantly superior outcomes correlates to less pronounced antibody responses in CF (167).

By investigating specific cytokine release from re-stimulated peripheral blood mononuclear cells (PBMCs), and later on cytokine measurements from unspecific stimulated T cells, a Th1/Th2 cytokine dichotomy in chronically infected CF patients was revealed (168, 169). Chronically infected CF patients had a Th2 dominated cytokine response with increased IL-4 (and IL-5, IL-10) production and diminished IFN-γ production. In addition, a similar Th1/Th2 cytokine dichotomy was later demonstrated in bronchoalveolar lavage fluid from subgroups of CF patients (170, 171). Interestingly, IFN-γ release from PBMCs correlated to an improved lung function, suggesting a potential beneficial effect of IFN-γ (168). Inbred mouse strains with chronic *P. aeruginosa* lung infection showed a pronounced pulmonary IFN-γ level in the relatively resistant C3H/HeN mouse (138, 172). Reinfection of the susceptible BALB/c mice resulted in a pulmonary Th1 response similar to the C3H/HeN mice and resembled the course of a primary infection in the C3H/HeN mice (173).

The explanation for the improved outcome of a Th1 dominated response in CF patients with chronic *P. aeruginosa* lung infection is incomplete, especially since the Th1 dominated response would be more appropriate towards intracellular pathogens. However, phagocytosis of apoptotic PMNs by alveolar macrophages before the PMNs progress into necrosis and thereby increase inflammation, is believed to be involved (174). Reduction of IL-8, the most important PMN chemoattractant is another likely mechanism (175, 176). A diminished Th2 response would presumably result in a reduced antibody response, due to reduced B and plasma-cell stimulation, and subsequently decreased immune complex formation and tissue damage.

Additional T cell subsets have been described, including the Th17 subset, characterized by production of IL-17 and sometimes IL-22 (177). Th17 cells are induced by TGF-β (178) and may be of interest in CF, since IL-17 induces the PMN mobiliser G-CSF and chemoattractant IL-8 (179, 180). In this way, Th17 may add to pulmonary pathology of chronic *P. aeruginosa* lung infections (179, 180). In sputum from stable CF patients and in chronically infected CF patients, IL-17 and IL-23, was increased as compared to CF patients without chronic *P. aeruginosa* lung infections (179). Interestingly, such difference was not observed in CF patients infected with *Staphylococcus aureus* (179). A substantially decreased fraction of peripheral Th17 cells in CF patients has been reported, and interpreted as augmented homing of the cells to the lungs, increasing the pulmonary inflammation (181). Determinations of cytokines related to Th subsets were conducted in children with CF, and demonstrated increase of both IL-17A and the Th2 related cytokines IL-5 and IL-13 in children with symptoms (180). In contrast, such relationship was not observed for Th1 related cytokines, indicating a correlation between Th2 and Th17 subsets in CF (180). Such a Th2-Th17 axis could dispose for *P. aeruginosa* lung infections, but this has not been clarified yet (171, 180, 182). Interestingly, T cell suppressive neutrophil myeloid-derived suppressor cells (MDSCs) has recently been reported in CF (183, 184). The presence of neutrophil MDSCs in peripheral blood correlated to improved lung function in CF in contrast to what would be expected (183). Down regulation of the harmful and dominating Th2 and Th17 response axis, could be the mechanism behind this observation.

The role of regulatory T cells (Treg), Th22, and additional T cell subsets has only been sparsely studied in biofilm infections. However, decreased levels and reduced functions of these immune cells in CF patients have been suggested and may result in augmented IL-17 and IL-8 production (182, 185).

## Novel Potential Treatment Options Towards *P. aeruginosa* Biofilm Infections

The administration of preformed antibodies or immunoglobulins to treat various infectious diseases is known as passive immunization therapy. Passive immunotherapy using avian IgY immunoglobulins (yolk) targeting *P. aeruginosa* represents an alternative to conventional antibiotic therapeutics. IgY is the predominant serum antibody in chickens and is the avian homologue of mammalian IgG (186). It accumulates in the egg yolk from the blood and provides the offspring with humoral immunity. Hyperimmunization of chickens with specific antigens, provides high yields of specific IgY antibodies in the egg yolk (187). *In vitro* studies with IgY targeting *P. aeruginosa* showed firm binding to flagella and interference with the adhesion of bacteria to epithelial cells (188). Potentially, such effect could prevent bacteria from colonizing the respiratory tract. Additionally, our group has also observed promising effects of anti-*P. aeruginosa* IgY. In *in vitro* studies, respiratory PMN burst and bacterial killing of *P. aeruginosa* were shown to be significantly increased in the presence of anti-*P. aeruginosa* IgY (189). Anti-*P. aeruginosa* IgY seems to affect aggregation of bacteria resulting in immobilization and increased surface hydrophobicity, enhancing non-Fc receptor mediated phagocytosis (190). The observed *in vitro* effects of anti-*P. aeruginosa* IgY, were in accordance with *in vivo* observations in an acute murine pneumonia model, where we demonstrated a 2-log reduction in pulmonary bacteria, which was paralleled by decreased inflammation in the airways of anti-*P. aeruginosa* IgY treated mice as compared to mice receiving non-specific IgY (191).

Potentially, anti-bacterial immunotherapies by means of pathogen specific IgY augments PMN mediated phagocytic effects and reduce the level of airway colonization in CF and may even potentiate the action of anti-pseudomonal antibiotics (192). Moreover, a clinical study examining the effects of oral prophylactic immunotherapy with anti-*P. aeruginosa* IgY in non-chronically infected CF patients has shown promising results (193).

Recombinant S100A8/A9 also show promising therapeutic properties. Our group found that immune modulation of *P. aeruginosa*-biofilm infected wounds on BALB/c mice by 4-days local application of recombinant S100A8/A9, combined with systemically administered ciprofloxacin, significantly reduced the bacterial load of the wounds (194). Since *in vitro* synergistic effect between S100A8/A9 and ciprofloxacin was not observed, the effect is highly dependent on host cells (194). Human studies and animal experiments indicate impairment of the S100A8/A9 response and that the level of S100A8/A9 is inappropriate in non-healing wounds. We are currently investigating this area to improve the understanding of the pathophysiological multifaceted role of S100A8/A9 in biofilm-infected wounds.

In adjunctive therapies of non-healing wounds with an inappropriate anti-biofilm host response, autologous fibrin rich patches containing thrombocytes and leucocytes are a promising treatment strategy (195). A three layered 3C patch, is produced by centrifugation of the patient’s whole blood in a specially developed device (195). The 3C patch is subsequently applied to the chronic wound (196). In an open study on chronic wounds of various backgrounds, an accelerated healing with 3C patches was revealed in the majority of the patients (197). The effect is most likely caused by production of healing growth factors and cytokines, e.g. PDGF-bb, from thrombocytes (195). In support of these observations, a substantial PMN activity was observed inside 3C patches in terms of respiratory burst, PMN phagocytosis activity and anti-biofilm action (196).

## Conclusions and Perspectives

Knowledge of the immune responses and bacterial defense mechanisms under conditions of biofilm infections is important as it constitutes an important part of the pathology of biofilm infections. As documented in the present review, our knowledge of immune responses to biofilm infections has increased considerably in recent years and is likely to provide important treatment tools against biofilm infections in the future. We may eventually be able to damping harmful immune system activities, or to activate parts of the immune system that can eradicate biofilm infections without causing detrimental collateral damage. In addition, antibiotic augmenting effects of the immune system could be identified. Alternatively, we may be able to manipulate the bacteria and down-regulate or eliminate the components of biofilms that are responsible for the recalcitrance towards immune system activities.

## Author Contributions 

All authors listed have made a substantial, direct and intellectual contribution to the work, and approved it for publication. All authors contributed to the article and approved the submitted version.

## Funding

This work was supported by a grant (DFF-7016-00039) to TT-N from the Danish Council for Independent Research. CM is supported by Novo Nordisk Fonden (Borregaard Clinical Scientist Fellowship in translational research; grant no. NNF17OC0025074).

## Conflict of Interest

The authors declare that the research was conducted in the absence of any commercial or financial relationships that could be construed as a potential conflict of interest.
